# Supplementary material for: Trait self-control does not predict attentional control: Evidence from a novel attention capture paradigm
Source: PLoS One. 2019 Dec 12;14(12):e0224882. doi: 10.1371/journal.pone.0224882 (PMC6907807; doi:10.1371/journal.pone.0224882)
Supplement: S1 Materials — (DOCX) [file pone.0224882.s005.docx]

**Supplementary Materials**

**Secondary Analyses**

In addition to our primary analyses, we ran secondary analyses to investigate attention capture effects in general. These analyses can be seen as manipulation checks. We conducted a two factor repeated measures ANOVA with the within-subject factors of cue match (match, mismatch) and cue side (same side, different side) on the following dependent variables: area under the curve, response time, initiation time, and time of maximum deviation. The results of these models are summarized in S1 Table.

Given that our primary interest was in trait self-control these analyses are not discussed in full detail. Here, we summarize the results and merely note that they follow the expected trends found in previous research (Dieciuc, Roque, & Boot, 2019). Area under the curve was greater for matching cues than mismatching cues and this difference was greater when the cue appeared on the opposite side of the target (Fig S1). Response time was greater for matching cues on the opposite side and mismatching cues on the same side than matching cues on the same side or mismatching cues on the opposite side (Fig S2). Initiation time was greater for mismatching cues than matching cues but this did not interact with cue side (Fig S3). Finally, time of maximum deviation was greater for mismatch same side cues than match same side cues but did not differ on the opposite side (Fig S4).

**Table S1**. **ANOVAs**.

|  | Dependent Variables | | | | | | | | | | | |
| --- | --- | --- | --- | --- | --- | --- | --- | --- | --- | --- | --- | --- |
|  | AUC | | | RT | | | IT | | | TMAD | | |
|  | F-stat | *p* | η_p_^2^ | F-stat | *p* | η_p_^2^ | F-stat | *p* | η_p_^2^ | F-stat | *p* | η_p_^2^ |
| Match | 25.12 | < .001 | 0.2 | 0.01 | 0.9 | < .001 | 41.61 | < .001 | 0.3 | 68.3 | < .001 | 0.41 |
| Side | 225.77 | < .001 | 0.7 | 27.56 | < .001 | 0.22 | 1.85 | 0.177 | 0.02 | 1.41 | 0.238 | 0.01 |
| Interaction | 192.41 | < .001 | 0.66 | 63.1 | < .001 | 0.39 | 3.79 | 0.054 | 0.04 | 45.52 | < .001 | 0.32 |

Two-factor ANOVAs for area under the curve (AUC), response time (RT), initiation time (IT), and time of maximum deviation (TMAD).

**Other Individual Differences**

Although we did not collect data on individual differences such as video game playing, which may have played a role in the capture of attention, we collected data on basic demographic variables, such as age, gender, and parents’ education and income. (Note that because our sample includes student participants, their educational attainment and income do not vary greatly.) Our primary concern is the association between trait self-control and individual differences in distractibility, namely, attention capture and disengagement. Hence, although we are not focused on the interaction between self-control and demographic variables here, it is important to control for them. Accordingly, we conducted correlation analyses controlling for these variables. Notably, this did not change the pattern of results that emerged without controls (S2 Table).

**Table S2. Correlations.**

|  | TSC | AUC_SELECTIVITY | AUC_DISENGAGE | TMAD_SELECTIVITY | TMAD_DISENGAGE |
| --- | --- | --- | --- | --- | --- |
| TSC | — |  |  |  |  |
| AUC_SELECTIVITY | .057 | — |  |  |  |
| AUC_DISENGAGE | .026 | .866*** | — |  |  |
| TMAD_SELECTIVITY | .121 | -.110 | .043 | — |  |
| TMAD_DISENGAGE | -.036 | -.643*** | -.684*** | .004 | — |

Pearson Correlations, controlling for age, gender, ethnicity, race, parental education, and parental income.

*p < .05, **p < .01, ***p < .001

**Table S3. Selectivity and Disengagement Correlations.**

|  | TSC | AUC_SELECTIVITY | AUC_DISENGAGE | TMAD_SELECTIVITY | TMAD_DISENGAGE |
| --- | --- | --- | --- | --- | --- |
| TSC | — |  |  |  |  |
| AUC_SELECTIVITY | .024 | — |  |  |  |
| AUC_DISENGAGE | -.010 | .864*** | — |  |  |
| TMAD_SELECTIVITY | .055 | -.119 | .040 | — |  |
| TMAD_DISENGAGE | -.037 | -.631*** | -.684*** | .020 | — |

Pearson Correlations amongst the various selectivity and disengagement scores. AUC = area under the curve. TMAD = time of maximum absolute deviation.

*p < .05, **p < .01, ***p < .001


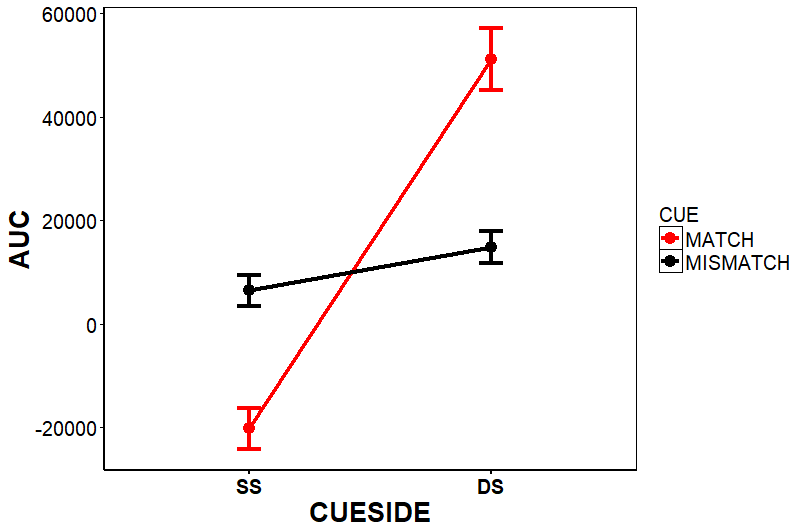


**Figure S1**. **Area under the curve**. Errors bars represent confidence intervals.


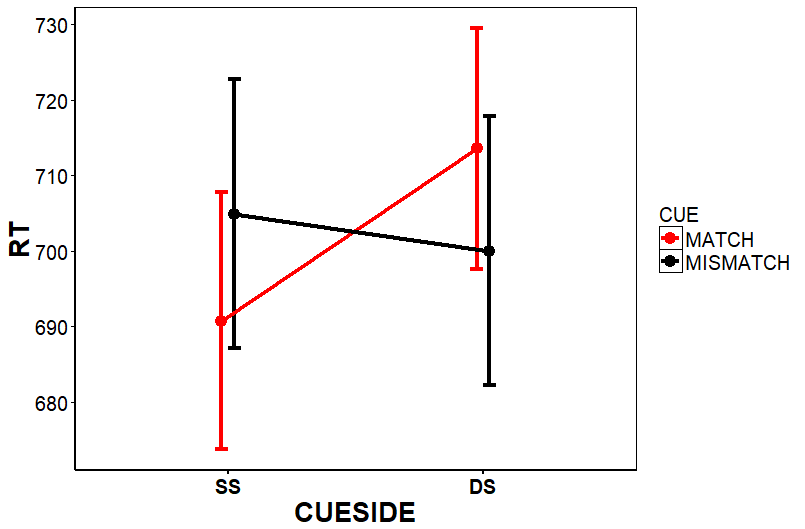


**Figure S2**. **Response time**. Errors bars represent confidence intervals.


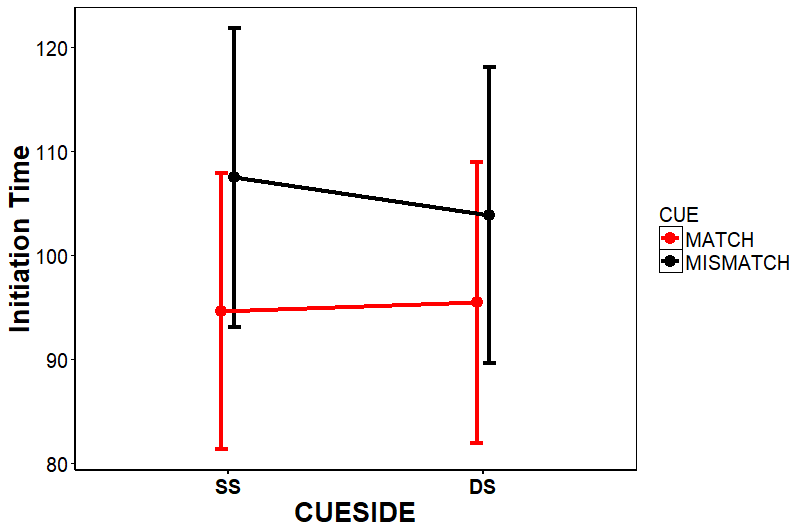


**Figure S3**. **Initiation time**. Errors bars represent confidence intervals.


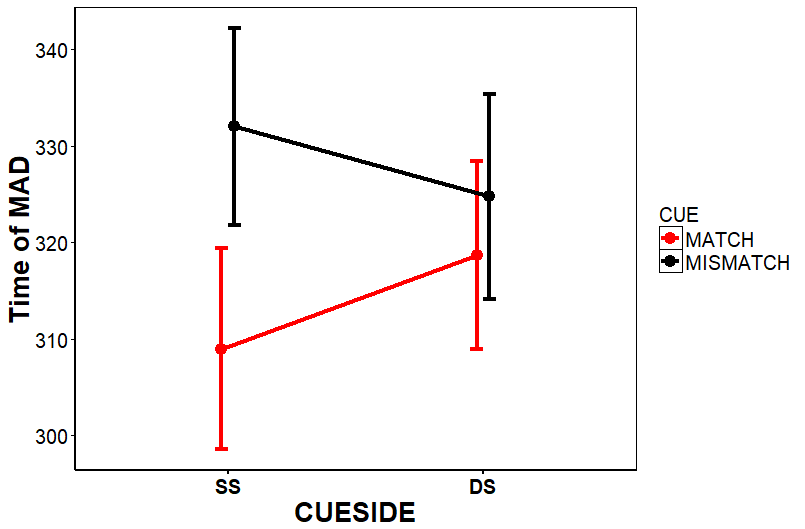


**Figure S4**. **Time of maximum absolute deviation**. Errors bars represent confidence intervals.
